# Characterization of multilevel influences of mental health care transitions: a comparative case study analysis

**DOI:** 10.1186/s12913-022-07748-2

**Published:** 2022-04-02

**Authors:** Kelsey S. Dickson, Marisa Sklar, Serena Z. Chen, Bo Kim

**Affiliations:** 1 Department of Child and Family Development, San Diego State University, San Diego, CA USA; 2 Child and Adolescent Services Research Center, San Diego, CA USA; 3 Department of Psychiatry, University of California, San Diego, San Diego, CA USA; 4 UC San Diego ACTRI Dissemination and Implementation Science Center, San Diego, CA USA; 5 Harvard South Shore Psychiatry Residency Training Program, Harvard Medical School / VA Boston Healthcare System, Boston, MA USA; 6 Department of Psychiatry, Harvard Medical School, Boston, MA USA; 7 Center for Healthcare Organization and Implementation Research, VA Boston Healthcare System, Boston, MA USA

**Keywords:** Mental health care transitions, Multiple case study, Children, Adults

## Abstract

**Purpose:**

Mental health care transitions are increasingly prioritized given their potential to optimize care delivery and patient outcomes, especially those focused on the transition from inpatient to outpatient mental health care. However, limited efforts to date characterize such mental health transition practices, especially those spanning multiple service setting contexts. Examination of key influences of inpatient to outpatient mental health care transitions across care contexts is needed to inform ongoing and future efforts to improve mental health care transitions. The current work aims to characterize multilevel influences of mental health care transitions across three United States-based mental health system contexts.

**Methods:**

A comparative multiple case study design was used to characterize transition practices within the literature examining children’s, non-VA adult, and VA adult service contexts. Andersen’s (1995) Behavioral Health Service Use Model was applied to identify and characterize relevant distinct and common domains of focus in care transitions across systems.

**Results:**

Several key influences to mental health care transitions were identified spanning the environmental, individual, and health behavior domains, including: community capacity or availability, cross-system or agency collaboration, provider training and experience related to mental health care transitions, client care experience and expectations, and client clinical characteristics or complexity.

**Conclusions:**

Synthesis illustrated several common factors across system contexts as well as unique factors for further consideration. Our findings inform key considerations and recommendations for ongoing and future efforts aiming to plan, expand, and better support mental health care transitions. These include timely information sharing, enhanced care coordination and cross setting and provider communication, continued provider/client education, and appropriate tailoring of services to improve mental health care transitions.

## Introduction

Mental health care systems have recognized the need to prioritize individuals’ care transition between health care levels and/or settings [1, 2]. Mental health conditions affect 46.6% of the U.S. population during their lives and 26.6% in any given year [3], often necessitating a higher level of care such as inpatient hospitalization. The number of inpatient hospitalizations related to mental health problems is growing, with a 17% increase from 2014 to 2018 and a larger increase than those observed for higher than non-mental health hospitalizations [4]. Further, the presence of mental health conditions, especially in youth, as well as those necessitating more intensive care such as inpatient care across the age span are associated with poor lifetime outcomes [5–8]. Together, these point to the need for targeted efforts to facilitate successful linkage between levels or types of care such as inpatient to outpatient care to prevent rehospitalization and improve outcomes for this high priority population. While care transition interventions, or those aimed at facilitating the transition from one type or level of care to another (e.g., emergency room to psychiatric inpatient unit, inpatient to outpatient), are being increasingly tested for general medical populations, few interventions specifically target mental health [9]. Those that do focus on mental health care transition practices are increasingly focused on improving inpatient to outpatient transitions [10, 11]. The current work aims to characterize multilevel influences of inpatient to outpatient mental health care transitions in service of informing ongoing and future efforts targeting improved mental health care transitions.

### Outcomes of poor care transitions

Poor transition between care levels or settings such as inpatient to outpatient are common and known to heighten risks of treatment disruption, nonadherence, service disengagement, hospital readmission, and worsening of symptoms [9, 12–14]. Connection to outpatient care within seven days of discharge is a widely accepted indicator of the quality of inpatient to outpatient transition; yet this connection is made for less than half of discharged patients within the United States (U.S.) [15]. Although initiation of, or initial attendance at, aftercare is a indicator of successful care transition, it is not a sufficient predictor of care transition outcomes. For example, among youth who make successful post-transition contact, limited engagement and attrition following initial or intake appointments is common [16]. Further, a poor transition experience is known to predict future care disengagement [12, 17]. Thus, successful transitions entail ongoing engagement and care utilization to optimize care outcomes for this population.

### The need for targeted mental health care transition efforts

There is a large literature underscoring the need for targeted transition practices, especially mental health focused practices, that meet individual needs and are synergistic with broader service systems (e.g., health care, educational, vocational) and health policy contexts [12, 17, 18]. Detailed within this literature are the multifaceted and multilevel, or those that span the multiple aspects of the care experience, determinants of transitions. Personal or patient level factors (e.g., care experience, attitudes, preparedness, knowledge or self-efficacy regarding mental health) greatly influence mental health care transitions [12, 17, 19]. Similarly, provider factors (e.g., provider training, experience, communication or collaboration practices) or broader service setting or context factors (e.g., differences in organizational culture or practices between care settings, existence of transition practices or processes, laws or funding, regional location) are all known to influence care transition [12, 20–22]. Importantly, these determinants are often dynamic, interactive, and contextualized. This is reflected in theories used to characterize health services utilization, including Andersen’s (1995) well-known Model of Behavioral Health Service Use and Munson and colleagues’ (2012) more recent Mental Health Service Utilization theory detailing the multilevel contextual factors, including both time variant and static factors, that influence mental health service among adults [23].

Despite the large extant literature focused on mental health transitions and pathways to care, much of this work focuses on examining transition to mental health more generally or transitions practices for specific diagnoses, age groups, or care contexts, with limited emphasis to date explicitly focused on the transition from inpatient to outpatient mental health care. In youth, for example, there are significant efforts to characterize determinants and improve the transition from child to adult mental health services [17, 19, 21, 24–26] as well as youth pathways to mental health care more generally (e.g. [27–29]). Those studies specifically focused on the transition from inpatient mental health care in youth largely target school transitions, with limited emphasis on the transition to non-school based mental health services [30, 31]. For adults, many of these studies focus on transitions between general medical settings, or transitions between mental health care settings [9]. While taking a whole-person orientation that is inclusive of an individual’s physical and mental health is encouraged [9, 32], few studies examine care transitions within the inpatient to outpatient mental health context span across broader multi-services settings contexts. Without such broader considerations, it is difficult to understand the relevant factors that impact transition practices overall, and also to determine ways in which transition practices in one context can be informed by efforts to account for particular relevant factors within other contexts.

### The current study

To start addressing this knowledge gap, this perspective piece examines examples of transitions in mental health care across types and/or levels of care such as inpatient to outpatient spanning three system contexts within U.S. Specifically, the child, adult [not specific to military veterans receiving care from the Department of Veterans Affairs (VA); henceforth “non-VA adult”], and adult VA service system contexts were targeted to identify characteristics of individual, provider, care setting, health care system, and policy-level considerations that shape their respective care transition practices. We chose these three health care system contexts to compare as they represent three major health care systems wherein a large proportion of the U.S. population engaged in mental health care receive treatment. Additionally, these contexts target substantially distinct populations, and are subject to different policies and structures. For example, the distributed provision of mental health care across multiple child-specific settings (e.g., education, child welfare) drives the different organizational or contextual factors influential of child compared to adult mental health care systems [33, 34]. For the adult population, the largely privatized health care system operates differently from the nationalized health care system within the VA [35]. The networks and policies that shape the private health care sector are distinct from those that impact the nationally integrated and publicly-funded VA system, and individuals receiving services from non-VA and VA systems differ significantly in both health (mental and physical) and socioeconomic status [36]. These differences between the child, non-VA adult, and VA adult mental health care render their contexts as meaningful comparative settings in which to examine diverse variations and overlaps in care transitions considerations across three systems that deliver mental health services to a nation’s population.

**Fig. 1:**
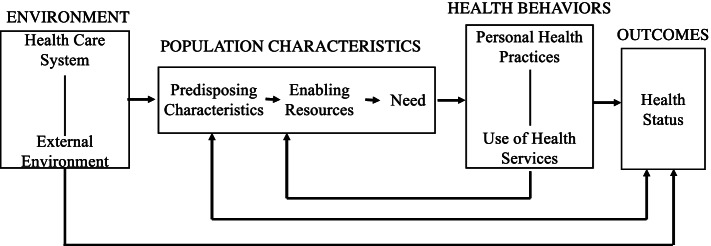
Anderson's (1995) model of behavioral health service

We structure this examination as a comparative multiple case study [37] of the different health system contexts, as curated by our team of authors who are active mental health care researchers, including with an emphasis on transition practices. This work’s aim was to characterize multilevel influences on transitions across mental health care type or levels by examining examples of transitions in different system contexts. The purpose of choosing a multiple case study approach was to generate an in-depth, multifaceted understanding of the multilevel influences on transitions. The nature of the inquiry was thus purposely broad, with the goal of serving as an initial step towards elucidating the directions that future investigations ought to pursue. Accordingly, the research aim called for observing the status of care transitions considerations across various settings and populations, addressable through the case study research method that focuses on exploring a contemporary phenomenon [37]. Therefore, we structured our work as a comparative multiple case study that follows a purposeful sampling strategy, which methodically draws on researcher expertise and experiences to select insightful cases that facilitate identification and understanding of commonalities and heterogeneities [38] regarding mental health care transitions across different system contexts. We restricted our focus to U.S.-based service settings within given the unique service context (e.g., presence of the V.A., non-centralized healthcare system); however, we feel this comparative case study has implications within the U.S. and beyond. Further, although it is outside the scope of the current review to include review the extant literature characterizing broader mental health care transitions (e.g., child to adult mental health services, general pathways to mental health care) given our explicit focus on inpatient to outpatient transitions, we refer this to help contextualize our current findings. We conceptually align the discussion to the Model of Behavioral Health Service Use (See Fig. 1) [39]. Specifically, we characterized distinct and common aspects of care transitions, as well as their causes (when available), across the different systems to domains detailed in the Behavioral Health Service Use Model. Based on noticed trends, we offer recommendations for the field on key considerations for efforts aiming to plan, expand, and better support mental health care transitions.

## Methods

We structured the examination of care transitions for mental health care across child, non-VA adult, and adult VA service system contexts as a comparative multiple case study [37]. Guided by the Behavioral Health Service Use Model [39] we focused on specifying the multiple influence domains on respective care transition practices, including system’s environment (e.g., health care system, external environment), population characteristics (e.g., predisposing characteristics, need), and health behaviors (e.g., personal health practices, service use). This model was selected given its utility and wide spread use to help frame and understand relevant factors of behavioral health service use. The Behavioral Health Service Use Model also details the interconnected nature of specified factors, which appropriately reflects the interrelatedness common among factors of care transition, further supporting our application of this model (See Fig. 1). We applied this model to guide service factor identification and reporting across the three service settings of interest. We also extended this model to account for additional ecological levels (e.g., community and policy, organizational, interpersonal) known to impact the services landscape [40]. Informed by current multiple case study methodology, we applied explicit study tactics toward establishing our specifications (identifying appropriate domains of focus, uncovering non-spurious relationships, defining the domain of generalizability, and ensuring the reliability of the study) through each of the data collection and data analysis phases of the study [37]. Additionally, we consulted health services experts with experience applying similar methodologies and leading similar reviews regarding our study design, fidelity to the comparative multiple case study approach, face validity of resulting interpretations, and approaches to further minimize author biases.

### Data collection

We employed the study tactic of identifying appropriate domains of focus by reviewing multiple sources of evidence. Guided by the Behavioral Health Service Use Model [39] and our collective research experience of the three mental health care systems, we first held a participant-facilitated discussion [37] to document our prior research-based knowledge under the domains of individual, provider, care setting, health care system, and policy-level considerations that shape each system’s care transition practices. Our participant-facilitated discussions consisted of self-facilitated discussions among the authors that followed established meeting facilitation strategies [41, 42]. Consistent with the comparative multiple case study steps of honing the (i) question, (ii) propositions, (iii) units of analysis, (iv) logic linking data to the propositions, and (v) criteria for interpreting the findings [37], these discussions were used to hone the appropriate domains of focus. We did so by brainstorming and reaching consensus to (i) purposively sample the literature to maximize variation in care transitions considerations across the three system contexts, (ii) consider the Behavioral Health Service Use Model’s domains and subdomains as elements to cover through the sampling, (iii) take individual published works to be the unit of analysis, (iv) align to the Behavioral Health Service Use Model to organize the collected data for explanation building, and (v) deliberate as a team to minimize individual author biases and maintain consistent data-to-interpretation across the three system contexts. To enable systematic documentation (and subsequent systematic analysis, as described below), we followed recommendations of Averill (2002)’s qualitative matrix analysis to organize the data from the discussion into an electronic spreadsheet-based matrix [43].

### Search and screening methods

We then conducted a literature review [37] of published studies that report on mental health care transition practices for each service system context, searching particularly for studies that provide supportive or alternative perspectives on how care transition-relevant domains were characterized as of the participant-facilitated discussion. We added insights from each reviewed study to the spreadsheet-based matrix, associating each entry with the domain (column) and source from which it originated (row). We utilized an iterative process whereby we completed a broad literature search that was supplemented by a more focused or targeted literature search following a review of initial findings and themes that emerged across our three contexts of interest. The initial broad literature search aided the generation of key search terms related to inpatient to outpatient mental health care transitions used for study identification via electronic database search in the targeted literature search. We identified additional studies through backward and forward searches in pertinent and seminal articles. Authors independently screened articles for inclusion. We included articles if they were written in English, conducted in the U.S., and reported on and pertained to one of the three service system contexts of interest in the current study.

### Data analysis

We employed the study tactic of uncovering non-spurious relationships by conducting pattern matching of collected data (i) across data sources for each mental health care system context and (ii) across care system contexts [37]. Using the data matrix, we conducted a three-step thematic analysis of prevalent trends in characteristics associated with each transition-relevant domain across the data sources and care systems:

1. We independently reviewed the data to identify emergent codes representative of the nature of the domains.
2. We conducted constant comparison combined with consensus-building discussions [37] to (i) refine the list of emergent codes and their definitions and (ii) assign finalized codes to the data.
3. We applied the Behavioral Health Service Use Model to formulate overarching themes based on reviewing the data associated with each code.

For results interpretation, we first continued the study tactic of uncovering non-spurious relationships by addressing potential rival explanations to our overarching themes. We held consensus-reaching discussions to specify the extent to which our collected and analyzed data are able to satisfactorily negate the rival explanations for each theme, methodically noting future investigations needed to support or alter the theme. We then defined the domain of generalizability by using replication logic involving the multiple sources from which the data originated. Namely, in interpreting the data, we kept in mind our targeted selection of data sources which could lead to illustrative inferences about key themes that are relevant to care transitions (and not statistical inferences about the identified themes’ prevalence) [44, 45].

To ensure the reliability of our data collection and analyses, we documented a detailed case study protocol and maintaining a thorough case study database. We organized the presentation of our findings by the specific corresponding component (in italics) as well as the broad domain (in parentheses) specified in the Behavioral Health Service Use Model–environment, population characteristics and health behavior. For each component, the Results section below delineates aspects of care transitions across the child/adolescent, non-VA adult, and VA mental health care system contexts, to provide cross-system knowledge that can inform future care transition planning.

## Results

### Children’s mental health service settings

Multiple factors emerged as relevant to transitions in children’s mental health care (see Table 1).

**Table 1:** Comparison of care transition factors across children’s, adult, and VA Mental Health (MH) service contexts

		Children’s MH	Non-VA Adult MH	VA MH
Domain	**Definition**			
**Environmental/Influence**				
External Environment				
1. Geographical Characteristics	1. Characteristics location of where services are provided (e.g., urban vs. rural area, average socioeconomic status of residents)	X	X	X
2. Community Capacity and Availability	2. Local community availability and offering and/or capacity to provide services, including alignment with insurance model	X	X	X
3. Service Provision Policy	3. Broader policies and legislation related to eligibility and service provision	X	X	X
4. Prioritization of Suicide Prevention	4. Local or broader prioritization of suicide prevention, both formally (e.g., policies, legislation) or informally (purveyor organizations, advocacy organizations)	X		X
5. Demand for MH Care	5. Current events driven changes in demand for mental health care (e.g. military conflicts)			X
Systems of Care				
1. Integration of Multiple Services Within and Across Sectors	1. The extent to which there is integration within and across multiple sectors of care (e.g., mental health and addiction, family and social services, corrections, education) serving the same patient population, in terms of policies, infrastructure, and processes.	X	X	X
2. Focus on Recovery	2. Prioritization or implementation of recovery-oriented care in addition to crisis services			X
3. Availability of Diverse Treatment Modalities	3. Availability and/or capacity to provide diverse services within the health care system		X	X
4. Within System Accessibility of Services	4. Accessibility of services between agencies or providers within a care system	X	X	X
5. Organizational Practice Culture	5. Organizational service provision or practice models or culture	X		X
**Population Characteristics**				
Provider Characteristics				
1. Training and Knowledge	1. Prior experience with formal or informal training and/or knowledge regarding mental health and mental health care transitions		X	X
2. Experience	2. Prior service provision experience including experience with various service types, agencies, an/or other providers and clients			X
3. Attitude and Beliefs	3. Attitudes and beliefs related to mental health and mental health service provision			X
4. Interprofessional Collaboration	4. Experience and/or preference specific to collaboration with other professionals and/or disciplines	X		X
Client/Patient Characteristics				
1. Functioning Level and Symptoms	1. Level of functioning, presenting symptoms, presence of co-occurring conditions, clinical complexity, and/or service needs	X	X	X
2. Race/Ethnicity	2. Race and/or ethnicity status	X	X	
3. Resources and/or Social Support	3. Available resources and/or supports (e.g., SES, social support networks)		X	X
4. Age	4. Age as it relates to receipt or access to services, including age-related transitions between systems of care	X	X	X
5. Gender	5. Gender as it relates to receipt or access to services			X
6. Homelessness	7. Housing status or access to housing		X	X
7. Involvement in multiple systems of care	8. Receiving care from, and which from more than one system of care	X	X	X
8. Insurance Status	9. Current insurance type, benefits, and/or status	X	X	
9. Attitudes and Beliefs	10. Attitudes and beliefs related to mental health and mental health services		X	
Caregiver Characteristics				
1. Caregiver Perceptions & Experience	1. Prior service provision experience with services, including various service types, agencies, providers	X		
2. Caregiver Psychosocial Factors	2. Factors related to functioning, symptoms and/or characteristics	X		
**Health Behavior**				
Client/Patient Health Practices				
1. Engagement in Care	1. History of and/or current engagement in services, including care attendance and participation	X	X	X
2. Medication Adherence/Compliance	2. Adherence or compliance to care recommendations and plans		X	X
3. Report of Symptoms	3. Tendency to under or over report current symptoms or functioning			X
4. Substance Use	4. Use of substances	X	X	X
5. Expectations or Readiness for Care	5. Expectation and/or readiness for care	X	X	X
Use of Health Services				
1. High Relapse Rates	1. Rates of relapse and impact on service needs and usage		X	X
2. Focused Care for Posttraumatic Stress Disorder	2. Availability/utilization of care specifically targeting trauma related symptoms			X

####  Environment/influence (external environment)

*Geographical characteristics* influence care transition, with residing in rural areas or those characterized by lower SES or medium to high unemployment rates demonstrated lower successful transition from inpatient care [46, 47]. Relatedly, *community capacity and availability* also impacts care transitions. Specifically, treatment in a psychiatric (versus general hospital) and higher volume of available general and specialty mental health providers such as psychiatry positively impacts aftercare or follow-up care decisions and usage while receiving care in larger healthcare facilitates and hospitals with higher Medicaid penetration negatively impacts follow-up [47]. The availability of peer specialists or peer support services associated within the mental health catchment area also positively impacts care transitions, with data noting higher outpatient care usage and lower inpatient usage among areas with peer support services [48]. In terms of *service provision policy* and *prioritization of suicide prevention*, the recent prioritization and funding calls to address suicide prevention, with an emphasis to prevent the need for crisis and/or intensive services, contributes to a larger focus on improving care transitions for youth [49, 50].

#### Intra-organizational or system characteristics (system of care environment)

Multiple service sectors and settings (e.g., educational, child welfare and juvenile justice) provide mental health care for youth [34], with most receiving multiple services spanning service sectors (e.g., school-based outpatient, medication management from pediatricians) [30]. The presence of an additional sector responsible for providing mental health services can impact successful care transition, including serving as a gatekeeper to services within and across sectors [33]. In particular, while these services can be linked (e.g., contracts to enable community outpatient clinics or providers to provide school-based services), there is often limited *integration of multiple services within and across sectors*, which can impede care transitions for youth. The siloed nature of care, which describe most states’ funding and governance structure for children’s services, directly contributes to communication and collaboration challenges across service settings [51]. This siloed care model also likely contributes to differing *organizational practice culture*, or beliefs and approaches to mental health treatment, that influence care transitions. For example, the culture within children’s mental health settings are often described as proactive, family oriented and inclusive that may be in contrast to the reactive, crisis management and primarily medical approach taken in hospital settings [25].

Both across and *within system accessibility of services*, issues related to infrastructure or capacity for cross organizational collaboration or communication also greatly impact care transition, including the ability to share medical or treatment records and coordinate care [52]. From a regulatory perspective, variations in relevant guidelines or policies (e.g., Health Insurance Portability and Accountability Act, Family Educational Rights and Privacy Act) significantly impact an organizations ability to share patient records across systems. Consistency in record management systems also impacts the logistics of record sharing, with common systems (e.g., sharing the same electronic health record system) aiding and differences (e.g., electronic versus paper) impeding this process.

#### Provider characteristics (presdisposing characteristics-provider)

Highlighting the role of *interprofessional collaboration*, shared goals, communication, and collaboration, or lack thereof, across service providers greatly impacts consistency and successful transition between levels of mental health care [52–55]. Recent findings suggest that communications between hospital staff and outpatient providers occur less than half the time; when it does occur, confusion or challenges regarding who to include in post-discharge communications and follow-up often excludes key providers from these communications [56].

####  Population characteristics (predisposing characteristics-client/patient)

 In terms of *client functioning level and symptoms*, higher client problem behaviors or family dysfunction as well as the presence of co-occurring chronic medical and substance use conditions limits successful transition and aftercare usage [47, 52, 57]. The presence of substance use disorders or developmental conditions such as autism spectrum disorder has unique implications for access and receipt of outpatient mental health care compared to inpatient psychiatric services, which greatly impacts aftercare options and availability [57–59]. This speaks to the impact of *involvement in multiple systems of care* and how diffusion of care often obfuscates the primary provider or system responsible for providing mental health care. Further, these challenges stem from the distributed or siloed nature of service and diffusion in care responsibilities across care sectors as well as local capacity and availability of appropriate care options.

Additional non-clinical factors are associated with post-hospitalization aftercare and outpatient service usage, such that older *age* and specific *insurance type* (e.g., having public versus private insurance) have implications for transitions [47, 57, 60]. Youth also face multiple age-related care transitions in care (e.g., from child to adult). Significant barriers and frustration surrounding this transition are well documented in the U.S., similar to those noted worldwide [17, 20] and serve to compound inpatient-to-outpatient care transitions, including issues related to eligibility and identification of outpatient services appropriate for youth during this transition time. Turning to *race/ethnicity*, youth from minority backgrounds are less likely to utilize and be referred to community-based or school-based mental health services, and often provided referrals for other treatment options such as residential treatment instead [46, 57, 61].

Unique to the child mental health setting, the presence of *caregiver related characteristics* impact children’s mental health care transitions. Caregiver perceptions regarding community social supports and current family functioning, prior experience and satisfaction with the children’s mental health and associated systems all influence a child’s care transitions [30, 62]. Caregiver psychosocial factors are also important, with caregiver strain, fewer mental health symptoms, higher sense of empowerment, coping and self-efficacy predictors of increased participation in care transition [30, 55].

#### Health behaviors (client/patient health practices)

Youth with prior history of *engagement in care*, especially a recent care episode, utilize more outpatient care use following an inpatient stay [46, 47, 57]. As alluded to above, a *history of substance use* is associated with a lower likelihood of successful access and receipt of outpatient mental health car [47, 57]. Although there is scant literature assessing youth expectations for care, *caregiver perceptions and/or expectations for care* are known predictors of care transitions, as discussed above.

### Non-VA adult mental health service settings

While numerous models exist for addressing transitions in care in the non-VA adult service system context, few have been reported that are specifically focused on transitions for the treatment of mental health concerns [63]. Rather, models for addressing transitions in care typically focus on the general medical patient population, and/or encourage care coordination efforts and transition efforts take a holistic approach to patient health and well-being that may include mental health together with general medical health. As a result, characteristics impacting transitions in care for mental health concerns in the non-VA adult service system context were often interwoven with multilevel factors influencing general medical health and healthcare (see Table 1).

####  Environment/influence (external environment)

Related to *geographical characteristics* and *community capacity and availability*, location differences and community characteristics pertaining to the availability of resources appeared most frequently [64]. As part of the Affordable Care Act, Congress directed the Centers for Medicare and Medicaid Services to penalize hospitals with poor 30-day readmission rates. This law triggered the investment of substantial resources in finding solutions to improving readmission rates. A growing body of evidence suggests that the primary drivers of variability in 30-day readmission rates are resources of the community in which the hospital is located [65]. The cost of care, distance from nearest provider, availability of providers accepting Medicare were frequently reported characteristics impacting transitions in mental health care [66–69]. The market share, or penetration, of health maintenance organizations in a defined community has a demonstrated relationship with transitions in care [69]. A positive relationship between health maintenance organization penetration on mental health service use among those with insurance has been found [70]. Geographic variations in the supply of providers have also been linked with long-term continuity of care [71].

#### Intra-organizational or system characteristics (systems of care environment)

For Medicare beneficiaries, psychiatric hospitals had lower follow-up rates than general hospitals, system-affiliated hospitals had lower follow-up rates than unaffiliated hospitals, and nonprofit hospitals had higher follow-up than public and for-profit hospitals [72]. Related to *integration of multiple services within and across sectors*, the presence of characteristics to facilitate coordinated and/or collaborative care within the health care system has also been linked to improved transitions in care [63, 73]. Core elements for a health care system include the use of data to identify patients at greatest risk, shared access to patient health records, authentic engagement of patient/family in treatment planning, clearly defined transition pathways, dedicated care staff to direct care transitions, and shared accountability for meeting quality expectations. The presence of discharge planning processes such that an initial, and timely, outpatient visit following discharge is scheduled has been associated with successful follow-up and reduced rehospitalization, even among patients at highest risk for unsuccessful care transitions [73–76]. The availability and provision of language-compatible services for non-English speakers has also been shown to predict service engagement including that following hospitalization [77]. Finally, the duration of case management intervention has been shown to predict successful care transitions such that shorter interventions served as a barrier to successful follow-up [75].

#### Population characteristics (predisposing characteristics-provider)

The literature regarding provider characteristics that influence transitions in mental health care in the non-VA adult service system context is sparse. However, *provider knowledge and understanding* has demonstrated impacts on transitions in mental health care. Care transition interventions had varying effectiveness dependent on the behaviors, opinions, affect, and education of staff delivering the interventions [73]. Provider competencies for navigating the service system and knowledge of complementary job tasks and functions of relevant providers involved in treatment can influence quality of care transitions [78, 79]. In addition to providing staff with adequate training on care transitions, willingness of staff to adapt and exhibit flexibility was also seen as key [73].

####  Population characteristics (predisposing characteristics-client/patient)

Regarding *functioning level and symptoms*, increased patient complexity and/or the presence of multimorbidities/medical comorbidities are commonly reported as negatively impacting transitions in mental health care [63, 80, 81]. *Age* influences transitions in care, with elder people being at higher risk of hospital readmissions than the general population [82]. Patient knowledge/awareness [63], as well as *attitudes and beliefs*, in particular stigma/negative beliefs about mental health [66, 83] predict transitions in mental health care. In terms of *resources and/or social support*, income [63], education [80], and employment [80] have all been shown to be positively related to follow-up care. *Racial and ethnic minorities* have poorer quality and access to behavioral health care even when they have adequate insurance [84]. Inconsistencies between studies were found regarding racial and/or ethnic differences in hospital readmission rates, though most show greater odds of readmission for white patients [85–87]. While many studies have shown a protective relationship between timely discharge with rehospitalization, this relationship does not exist for *homeless* patients [74]. Thirty day follow-up rates post-discharge among patients insured with Medicare is approximately 56% compared to 77% among patients with commercial *insurance* plans [88]. A history of *mental health service use* was another significant predictor of follow-up [80]. Hospital length of stay was also negatively related to readmission, such that there was a protective role of length of stays higher than 28 days [64, 85, 89].

#### Health behavior (client/patient health practices)

Little research exists regarding patient health behaviors that impact transitions in care for non-VA adults. Some studies, however, have found a negative effect of active *substance use* at time of hospitalization on likelihood of attending outpatient treatment [87, 90].

### VA mental health service settings

See Table 1 for care transition factors identified for VA mental health service settings.

#### Environment/influence (external environment)

Care transitions can be challenging for rural veterans (*geographical characteristics*) who commonly utilize non-VA hospitals [91], as non-VA hospitals may be less familiar with linkages that meet veteran-specific needs and preferences. Post-transition availability and access to needed resources and services (*community capacity and availability*) influence care transitions. Availability of post-transition self-management resources often varies across communities [92], and when available, peer support can be a valuable resource [93]. Linkages to community resources can be useful for veterans who disengage from post-transition care [94]. Also in terms of *service provision policy*, policies regarding veterans’ military service-connected eligibility for receiving VA care affects which services are accessible to them [95]. Beyond individual communities within which VA care settings exist, nationwide focus on veteran *suicide prevention* has identified how transitions and recovery approaches support suicide prevention [96]. Additionally, high and increasing *demand for mental health care* makes improving access a central VA goal [97].

#### Environment/influence (systems of care)

For *integration of multiple services within and across sectors*, challenges include continued practice of traditional (i.e., less recovery-focused) care [96] and inconsistent information transfer between settings [98]. Transition programs that *focus on recovery* are being tested and used [99, 100], and VA has endorsed an organization-wide change of mental health care to more explicitly reflect recovery values [93]. Furthermore, with regard to recovery-oriented services, each of Freedom Commission’s recommendations, SAMHSA’s framework, and policy directives on such services urges the need for transitions that specifically support recovery [101]. As an organization, VA has been focusing on creating and maintaining a portfolio of *diverse treatment modalities* to meet the needs of veterans who transition between care settings [102]. The coordination needed for care transitions and ongoing management largely utilize VA’s electronic health record, which is facing an imminent change to a new platform; this change’s implications for transition management are unclear [99].

Practitioners sometimes disagree whether increasing transitions increases admissions [103], especially when post-transition treatment cannot be arranged [104] due to limited *accessibility of services*. Moreover, different VA clinics handle transitions differently (e.g., continuity of care, hospitalist environment) [105]. This variability, which drives and is driven by varied *organizational cultures*, makes difficult widely applicable policies to incentivize appropriate transitions [106]. Coordination for transitions is further challenged for populations of veterans that interact with systems outside of health care. For instance, for justice-involved veterans, transition planning often requires medication coordination and records transfer across correctional and health care systems [107].

#### Population characteristics (predisposing characteristics-provider)

Decisions and common practices (e.g., appropriate length of inpatient stay, criteria for discharge) vary widely across providers [99], possibly due to limited standardized *training and knowledge *[98]. And perhaps as a related matter, use of measurement-based care approaches to inform transition-related care decisions is not yet common among providers [108]. Post-transition monitoring requires both provider *experience* or expertise and resources [109]. These are challenging when needs differ across veterans (e.g., different disorders) [110]. Differences in provider *attitude and beliefs* may heighten these challenges, even though it is not uncommon for *interprofessional collaboration* to be sought to coordinate and prepare veterans’ transitions [96].

#### Population characteristics (predisposing characteristics-client/patient)

For *functioning level and symptoms*, comorbidities are prevalent [92], making transition planning challenging [99]. Awareness and knowledge of available *resources and/or support* (e.g., community-based resources, crisis intervention support) has long been found to be helpful for post-transition care [111]. The client landscape is undergoing change as there is an increasing number of younger veterans (*age*) from recent wars and an increasing number of women veterans (*gender*) [103]. Also, with an estimated 37,000 veterans experiencing homelessness [112], the need is high for *homelessness* support following transition [103]. Post-transition care is particularly challenging for homeless veterans, with whom it is difficult for the health care system to maintain consistent and frequent contact [113]. In addition, veterans who are *involved in multiple systems* of care through being dually both enrolled in the VA health care system and also receive non-VA medical care (e.g., through Medicare coverage) are deemed particularly at risk for care that is not well coordinated [114], and this poses challenges for when a care transition involves the additional dimension of transitioning across care system boundaries.

#### Health behavior (client/patient health practices)

Relative to the non-VA adult population, the veteran population is characterized by prevalent poor treatment *engagement *[102]. Not too dissimilarly to other patient populations, *medication adherence* and compliance are common challenges across care setting boundaries [94]. The subjective nature of *reporting of symptom*s and how symptom severity is reported [99] often makes care planning more difficult for this population than it already is, especially considering the prevalence of comorbidities as mentioned above (including prevalence of *substance use* disorders). Most notably, veterans’ *expectations* of treatment, including what care and associated timely transitions consist of, may not always be in line with the recovery-oriented approach that encourages change, and this can impact the veterans’ level of investment in putting forth efforts to fully capitalize on their treatment and make notable and measurable improvements in their mental health [115].

#### Health behavior (use of health services)

The veteran population is characterized by *higher relapse rates* relative to the non-VA adult population [102]. *Posttraumatic stress disorder* among the population continues to be an emphasis and prevalent condition that requires focused care [101]. Also, care decisions (e.g., for transitions between care settings) are at times impacted by consideration of potential aggressive behavior by the military-trained veteran population [116].

## Discussion

**Fig. 2:**
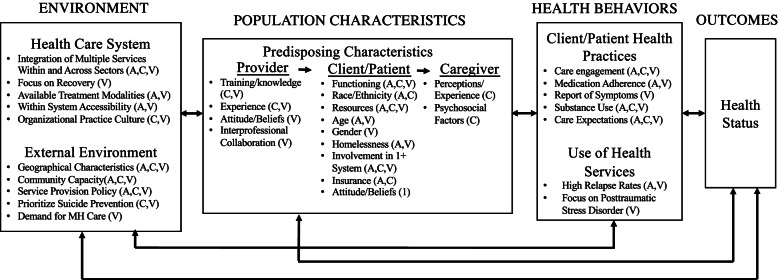
Model of mental health care transition determinants. This model builds of Anderson's model of behavioral health service use populated with findings from the current comparative case study to build and understanding of key factors impacting mental health care transitions. Note: C = Children's, A = Non-VA Adult; V = VA Adult

Our understanding of mental health care transitions thus far has been informed mostly by either (i) general and high-level concepts or (ii) non-comparative context-specific knowledge and perspectives. Through comparative multiple case study design, we applied the Behavioral Health Service Use model to examine mental health care transitions, especially inpatient to outpatient transitions, across child, non-VA adult, and VA service system contexts and identify multilevel factors or considerations that shape their respective care transition practices (see Fig. 2). We characterized a series of distinct and common aspects of care transitions across three key mental health service system contexts (see Table 2). This enabled explicit recommendations for the field on both strong practices and improvement opportunities, based on experiences and lessons that can be shared across the different systems. We also refer to the existing literature characterizing further mental healthcare transitions such as those from the child to adult system and initial pathways to care to help contextualize our findings and guide recommendations.

**Table 2:** Key factors and areas for further consideration identified

Environment	KEY FACTORS: • Existing community capacity • Infrastructure to support cross system or agency communication and collaboration FOR FURTHER CONSIDERATION: • Care coordination processes across agencies or sectors • Variability between organizations or sectors • Emphasis on whole-person based care models
Population	KEY FACTORS: • Provider training and experiences specific to mental health care transitions • Client clinical presentation and complexity FOR FURTHER CONSIDERATION • Caregiver experience and psychosocial factors • Additional provider factors known to impact care implementation • Societal identity
Health Behavior	KEY FACTORS: • History of care engagement and use, including adherence or compliance • Expectations or readiness for care • Additional health behaviors (e.g., substance use) FOR FURTHER CONSIDERATION: • Focused care • Report of symptoms and impact on broader care provision

### Environmental findings and implications

Our environment-level findings highlighted the importance of community capacity or availability and cross-system or agency collaboration, which is consistent with broader literature noting the facilitative role of effective transition-focused communication, processes or procedures during the transition from child to adult care systems [21] as well as that highlighting the key role of broader system capacity and factors connecting or linking multiple contexts in the implementation process [117, 118]. Fortunately, collaboration is encouraged through heightened national prioritization of integrated care and care coordination/navigation [119, 120]. There is also work highlighting or providing guidance on new or emerging methodologies as well as growing real-world case examples to inform future efforts that, in combination with national prioritization, will greatly facilitate increased community capacity and collaboration [121–123]. These findings have a number of implications for improving mental health care transitions. At the broader system or policy level, this may include the prioritization of improving community capacity and availability of resources and interventions within and across systems and developing or maintaining infrastructure to support communication and collaboration. This could include, for example, requirements surrounding ongoing collaboration between sectors and/or organizations or the provision resources and funding focused on developing or expanding transition specific programs.

In addition to policy efforts, care organizations can take several important steps towards improving capacity and collaboration. The creation of an organizational climate or culture engendered towards whole-person care may result in the actual delivery of whole-person care [124–126]. This includes creation or prioritization of care-coordination processes through embedding specified patient navigators or workflows designed to optimize inter and intra agency collaboration. Consideration and development of communication infrastructure and processes are also key within and between organizations, including well-explicated processes for the transfer of medical record information. For providers, engendering a practice with collaboration and open communication with other providers is a central component will significantly support the care transition process. Such processes and practices are considered relevant factors for promoting interagency collaboration and successful care transitions [21, 120] and consistent with findings noting the importance of a champion in the promotion of new care practices [127]. Finally, for clients, this may include a request for collaboration among treatment teams and organizations.

### Population level findings and implications

Our findings pertaining to population and health behavior results also have key policy and practice implications. Specifically, our findings surrounding importance of prior care experience or training, including that pertaining to mental health care generally and that specific to care transitions, highlight the importance of prioritizing educational opportunities for providers, caregivers and individuals to optimize the care transition process. This is consistent with data noting the influence of prior care experiences and training in transition practices on other types of mental health care transitions [22, 29]. Across the system or policy and organizational levels, this could include prioritization or funding enabling continued provider education regarding evidence-based care transitional practices and provision of educational outreach aimed at promoting knowledge and competency in mental health transitions [128, 129]. For clients, education can be used to promote health practices that support well-being [130, 131] and setting expectations regarding care transitions’ impact on health services use can help mitigate both patients’ and providers’ concerns surrounding transitioning to lower-intensity levels of care [132].

Our population-level findings also underscored the impact of client clinical complexity and the presence of co-occurring conditions on care transitions, mirroring prior findings noting challenges in mental health care transitions for those with co-occurring or complex mental health needs [17, 133]. There are several considerations relevant at the system and policy level. Specifically, reconsideration of current care provision policies, especially those specific to individuals with co-occurring conditions, may be needed to promote better care transitions for this population. For both care systems and organizations, prioritization and implementation of effective strategies targeting appropriate care transitions are needed. Fortunately, there is growing research delineating appropriate strategies to provide individualized and tailored care transitions for those with complex clinical profiles [133–135]. For providers or clients, efforts to increase awareness and/or collaboration with existing programs providing care transitions can be a great step forward towards improving care transitions for individuals with complex or co-occurring conditions.

Finally, our findings underscore the interconnected and bidirectional associations between the multilevel domains and subsumed characteristics (see Fig. 2). For example, the impact of prior care utilization and engagement within the health domains on care transitions is intricately linked with broader population characteristics (e.g., client functioning, insurance) as well as the broader external environment factors (e.g., community capacity, siloed nature of care). This is consistent with the broader literature noting the multilevel and bidirectional factors impacting the services landscape [40]. In turn, this highlights the need and utility of considering multiple domains of influence in efforts to improve mental health care transitions.

### Limitations

Our findings and recommendations outlined above should be considered with regards to three main limitations of this work that are worth noting. First, this work stems from our author team’s collective knowledge and experience of transitions across care types or levels such as inpatient to outpatient in the three different U.S.-based systems examined, and is thus based on a targeted but non-systematic review of the literature. It is therefore possible that there are examples of care transition-related efforts that have not been included in this examination, which could potentially alter our results. However, we adhered to established strategies for multiple case study designs. For example, we applied sampling criterion to add to our purposeful sampling strategy of maximum variation, which targets cases that, “despite having diverse variations, exhibit important common patterns that cut across variations” [38]. Relatedly and as briefly discussed above, it is also important to note that additional care transition factors likely exist but are not represented or disseminated in the academic literature. As such, there is a need for the field to be mindful of possible further associations and strongly recommend further examination and measurement of key factors identified as part of community or practice-based efforts surrounding care transition.

Second, this work focuses on describing the commonalities and heterogeneities of three service system contexts, each of which can arguably be subdivided into finer categories of populations and systems that are worthy of examination and comparison to one another. Rather than considering this work to be not applicable to such different potential categorizations of existing mental health care populations and systems, we believe that this work can provide a framework by which future investigations of care transition factors can be conducted for differently defined categories of mental health care populations and systems that take part in care transitions.

Third, this work did not characterize or compare the outcomes associated with the examined care transition factors. This was a deliberate design decision on our part to prevent our work from inappropriately under- or over-stating the potential comparative effectiveness of care transition practices that were reported by our included examples, given the diverse populations and subpopulations of concern across the different examples. Accordingly, meaningful future research building on this work would be to conduct cross-system interventional investigations of novel care transition practices that both (i) account for common considerations and (ii) adjust for context-specific considerations that are recommended by this work.

## Conclusions

Mental health care is increasingly viewed as an integral part of addressing whole-person needs, and this calls for transitions of mental health care to be carried out in a way that adequately accounts for individual needs, preferences, and characteristics. For the care transition factors related to environment/influence, population characteristics, and health behavior, this work accordingly highlights the importance of (i) timely information sharing, (ii) enhanced care coordination and communication channels, (iii) continued provider/client education, and (iv) appropriate tailoring of services regarding transitions between levels of care for mental health. Future research stemming from this work can focus on comparing this work’s U.S.-based findings to care transition considerations of other countries, tracking changes in prevalent themes over time (e.g., aligned to changes in local/regional/national health care policies), and/or conducting trials of transition across care levels or types interventions that examine the impact of enhancing usual care in one or more of the dimensions highlighted in this work. Resulting advances in knowledge and evidence regarding mental health care transitions will be relevant across diverse health care contexts that prioritize the delivery of high-quality care across different settings.

## Data Availability

All data generated or analyzed during this project are included in this published article.

## Abbreviations

U.S.: United States
VA: Department of Veterans Affairs

## References

[1] Kurdyak P, Vigod SN, Newman A, Giannakeas V, Mulsant BH, Stukel T (2018). Impact of Physician Follow-Up Care on Psychiatric Readmission Rates in a Population-Based Sample of Patients With Schizophrenia. Psychiatric Serv.

[2] Shaffer SL, Hutchison SL, Ayers AM, Goldberg RW, Herman D, Duch DA, Terhorst L (2015). Brief Critical Time Intervention to Reduce Psychiatric Rehospitalization. Psychiatric Serv.

[3] Kessler RC, Wang PS (2008). The Descriptive Epidemiology of Commonly Occurring Mental Disorders in the United States. Annu Rev Public Health.

[4] Health Care Cost Institute. 2018 Health Care Cost and Utilization Report. 2020 Retrieved from website: https://healthcostinstitute.org/images/pdfs/HCCI_2018_Health_Care_Cost_and_Utilization_Report.pdf.

[5] Copeland WE, Shanahan L, Hinesley J, Chan RF, Aberg KA, Fairbank JA, Costello EJ (2018). Association of Childhood Trauma Exposure With Adult Psychiatric Disorders and Functional Outcomes. JAMA Netw Open.

[6] Davis M, Banks S, Fisher W, Grudzinskas A (2004). Longitudinal patterns of offending during the transition to adulthood in youth from the mental health system. J Behav Health Serv Res.

[7] Pelham WE, Page TF, Altszuler AR, Gnagy EM, Molina BSG, Pelham WE (2020). The long-term financial outcome of children diagnosed with ADHD. J Consult Clin Psychol.

[8] Greenberg GA, Rosenheck RA (2005). Special Section on the GAF: Continuity of Care and Clinical Outcomes in a National Health System. Psychiatric Serv.

[9] Bao Y, Casalino LP, Pincus HA (2012). Behavioral Health and Health Care Reform Models: Patient-Centered Medical Home, Health Home, and Accountable Care Organization. J Behav Health Serv Res.

[10] Guzman-Parra J, Moreno-Küstner B, Rivas F, Alba-Vallejo M, Hernandez-Pedrosa J, Mayoral-Cleries F (2017). Needs, Perceived Support, and Hospital Readmissions in Patients with Severe Mental Illness. Commun Ment Health J.

[11] von Wyl A, Heim G, Rüsch N, Rössler W, Andreae A. Network coordination following discharge from psychiatric inpatient treatment: a study protocol. BMC Psychiatry, 2013;13(1). 10.1186/1471-244x-13-220. 10.1186/1471-244X-13-220 PMC3846805 24007198

[12] Appleton R, Connell C, Fairclough E, Tuomainen H, Singh SP (2019). Outcomes of young people who reach the transition boundary of child and adolescent mental health services: a systematic review. Eur Child Adolesc Psychiatry.

[13] Bartels SJ (2003). Improving the United States’ System of Care for Older Adults With Mental Illness: Findings and Recommendations for The President’s New Freedom Commission on Mental Health. Am J Geriatric Psychiatry.

[14] Horvitz-Lennon M, Kilbourne AM, Pincus HA (2006). From Silos To Bridges: Meeting The General Health Care Needs Of Adults With Severe Mental Illnesses. Health Aff.

[15] National Committee for Quality Assurance. State of Health Care Quality. 2011 Retrieved from https://www.ncqa.org/report-cards/health-plans/state-of-health-care-quality-report/.

[16] Becker KD, Boustani M, Gellatly R, Chorpita BF (2017). Forty Years of Engagement Research in Children’s Mental Health Services: Multidimensional Measurement and Practice Elements. J Clin Child Adolesc Psychol.

[17] Singh SP (2009). Transition of care from child to adult mental health services: the great divide. Curr Opin Psychiatry.

[18] Hansen EB, Seybolt DC, Sundeen SJ (2014). State Mental Health Policy: Building a Successful Public-Academic Partnership to Support State Policy Making. Psychiatric Serv.

[19] Butterworth S, Singh SP, Birchwood M, Islam Z, Munro ER, Vostanis P, Khan A, Simkiss D (2017). Transitioning care-leavers with mental health needs: ‘they set you up to fail!’. Child Adolesc Mental Health.

[20] Embrett MG, Randall GE, Longo CJ, Nguyen T, Mulvale G (2015). Effectiveness of Health System Services and Programs for Youth to Adult Transitions in Mental Health Care: A Systematic Review of Academic Literature. Adm Policy Mental Health Mental Health Serv Res.

[21] Livanou M, D’Souza S, Lane R, La Plante B, Singh SP. Challenges and facilitators during transitions from adolescent medium secure units to adult services in England: interviews with mental healthcare professionals. Administration Policy Mental Health Mental Health Services Res. 2021;1–16. 10.1007/s10488-021-01115-9 PMC8502166 33625622

[22] Russet F, Humbertclaude V, Dieleman G, Dodig-Ćurković K, Hendrickx G, Kovač V, Purper-Ouakil D (2019). Training of adult psychiatrists and child and adolescent psychiatrists in Europe: a systematic review of training characteristics and transition from child/adolescent to adult mental health services. BMC Med Educ.

[23] Munson MR, Jaccard J, Smalling SE, Kim H, Werner JJ, Scott LD (2012). Static, dynamic, integrated, and contextualized: A framework for understanding mental health service utilization among young adults. Soc Sci Med.

[24] Cleverley K, Rowland E, Bennett K, Jeffs L, Gore D (2020). Identifying core components and indicators of successful transitions from child to adult mental health services: a scoping review. Eur Child Adolesc Psychiatry.

[25] McLaren S, Belling R, Paul M, Ford T, Kramer T, Weaver T, Singh SP (2013). “Talking a different language”: an exploration of the influence of organizational cultures and working practices on transition from child to adult mental health services. BMC Health Serv Res.

[26] Singh SP, Tuomainen H, Bouliotis G, Canaway A, De Girolamo G, Dieleman GC. … MILESTONE Consortium. Effect of managed transition on mental health outcomes for young people at the child–adult mental health service boundary: a randomised clinical trial. Psychol Med. 2021;1–12. 10.1017/S0033291721003901 PMC10123823 37310306

[27] Iyer SN, Shah J, Boksa P, Lal S, Joober R, Andersson N, Malla AK (2019). A minimum evaluation protocol and stepped-wedge cluster randomized trial of ACCESS Open Minds, a large Canadian youth mental health services transformation project. BMC Psychiatry.

[28] MacDonald K, Fainman-Adelman N, Anderson KK, Iyer SN (2018). Pathways to mental health services for young people: a systematic review. Soc Psychiatry Psychiatr Epidemiol.

[29] MacDonald K, Ferrari M, Fainman-Adelman N, Iyer SN (2021). Experiences of pathways to mental health services for young people and their carers: a qualitative meta-synthesis review. Soc Psychiatry Psychiatr Epidemiol.

[30] Blizzard AM, Weiss CL, Wideman R, Stephan SH (2016). Caregiver Perspectives During the Post Inpatient Hospital Transition: A Mixed Methods Approach. Child Youth Care Forum.

[31] White H, LaFleur J, Houle K, Hyry-Dermith P, Blake SM (2017). Evaluation of a school-based transition program designed to facilitate school reentry following a mental health crisis or psychiatric hospitalization. Psychol Sch.

[32] Settipani CA, Hawke LD, Cleverley K, Chaim G, Cheung A, Mehra K, Henderson J (2019). Key attributes of integrated community-based youth service hubs for mental health: a scoping review. Int J Mental Health Syst.

[33] Garland AF, Hough RL, Landsverk JA, Brown SA (2001). Multi-Sector Complexity of Systems of Care for Youth With Mental Health Needs. Children’s Serv.

[34] Duong MT, Bruns EJ, Lee K, Cox S, Coifman J, Mayworm A, Lyon AR (2021). Rates of Mental Health Service Utilization by Children and Adolescents in Schools and Other Common Service Settings: A Systematic Review and Meta-Analysis. Adm Policy Mental Health Mental Health Serv Res.

[35] Shulkin DJ (2016). Why VA Health Care Is Different. Federal practitioner: for the health care professionals of the VA. DoD and PHS.

[36] Jha AK (2016). Learning From the Past to Improve VA Health Care. JAMA.

[37] Yin R (2018). Case Study Research and Applications: Design and Methods.

[38] Patton MQ (1990). Qualitative Evaluation and Research Methods.

[39] Andersen RM (1995). Revisiting the Behavioral Model and Access to Medical Care: Does it Matter?. J Health Soc Behav.

[40] Aarons GA, Hurlburt M, Horwitz SM (2010). Advancing a Conceptual Model of Evidence-Based Practice Implementation in Public Service Sectors. Adm Policy Mental Health Mental Health Serv Res.

[41] Bens I (2005). Advanced Facilitation Strategies. Tools & Techniques to Master Difficult Situations.

[42] Hogan C (2009). Understanding Facilitation. Theory and Principles.

[43] Averill JB (2002). Matrix Analysis as a Complementary Analytic Strategy in Qualitative Inquiry. Qual Health Res.

[44] Wood M, Christy R (1999). Sampling for Possibilities. Qual Quant.

[45] Chang Y, Voils CI, Sandelowski M, Hasselblad V, Crandell JL (2009). Transforming Verbal Counts in Reports of Qualitative Descriptive Studies Into Numbers. West J Nurs Res.

[46] Bridge JA, Marcus SC, Olfson M (2012). Outpatient Care of Young People After Emergency Treatment of Deliberate Self-Harm. J Am Acad Child Adolesc Psychiatry.

[47] Fontanella CA, Hiance-Steelesmith DL, Bridge JA, Lester N, Sweeney HA, Hurst M, Campo JV (2016). Factors Associated With Timely Follow-Up Care After Psychiatric Hospitalization for Youths With Mood Disorders. Psychiatric Serv.

[48] Ojeda VD, Jones N, Munson MR, Berliant E, Gilmer TP (2021). Roles of peer specialists and use of mental health services among youth with serious mental illness. Early Interv Psychiat.

[49] National Institute of Mental Health. RFA-MH-21-110: Service-Ready Tools for Identification, Prevention, and Treatment of Individuals at Risk for Suicide (R01 Clinical Trial Optional). 2020. Retrieved March 25, 2021, from grants.nih.gov website: https://grants.nih.gov/grants/guide/rfa-files/RFA-MH-21-110.html.

[50] National Action Alliance for Suicide Prevention. U.S. National Suicide Prevention Research Efforts: 2008–2013 Portfolio Analyses. In theactionalliance.org. 2015. Retrieved from https://theactionalliance.org/resource/us-national-suicide-prevention-research-efforts-2008-2013-portfolio-analyses.

[51] Davis M, Sondheimer DL (2005). State child mental health efforts to support youth in transition to adulthood. J Behav Health Serv Res.

[52] Clemens EV, Welfare LE, Williams AM (2011). Elements of successful school reentry after psychiatric hospitalization. Preventing School Failure: Alternative Educ Children Youth.

[53] Marraccini ME, Lee S, Chin AJ (2019). School reintegration post-psychiatric hospitalization: protocols and procedures across the nation. School mental health.

[54] Savina E, Simon J, Lester M (2014). School Reintegration Following Psychiatric Hospitalization: An Ecological Perspective. Child Youth Care Forum.

[55] Weiss CL, Blizzard AM, Vaughan C, Sydnor-Diggs T, Edwards S, Stephan SH (2015). Supporting the transition from inpatient hospitalization to school. Child and Adolescent Psychiatric Clinics.

[56] Simon JB, Savina EA (2010). Transitioning Children from Psychiatric Hospitals to Schools: The Role of the Special Educator. Residential Treat Child Youth.

[57] Fontanella CA, Warner LA, Steelesmith DL, Brock G, Bridge JA, Campo JV (2020). Association of Timely Outpatient Mental Health Services for Youths After Psychiatric Hospitalization With Risk of Death by Suicide. JAMA Netw Open.

[58] Brookman-Frazee L, Baker-Ericzén M, Stadnick N, Taylor R. Parent Perspectives on Community Mental Health Services for Children with Autism Spectrum Disorders. J Child Fam Stud. 2012; 21(4), 10.1007/s10826-011-9506-8. 10.1007/s10826-011-9506-8 PMC3826258 24244083

[59] Hawkins EH (2009). A Tale of Two Systems: Co-Occurring Mental Health and Substance Abuse Disorders Treatment for Adolescents. Ann Rev Psychol.

[60] Lynch S, Witt W, Ali MM, Teich J, Mutter R, Gibbons B, Walsh C. Follow-Up Care After Behavioral Health-Related Hospitalization for Children and Adolescents. Commun Mental Health J. 2020;56(8). 10.1007/s10597-020-00585-9 32072374

[61] Yeh, M., McCabe, K., Hurlburt, M., Hough, R., Hazen, A., Culver, S., … Landsverk, J. Referral sources, diagnoses, and service types of youth in public outpatient mental health care: A focus on ethnic minorities. J Behav Health Serv Res 2002;29(1), 45–60. 10.1007/bf02287831. 10.1007/BF02287831 11840904

[62] Smith T, Linnemeyer R, Scalise D, Hamilton J (2013). Barriers to Outpatient Mental Health Treatment for Children and Adolescents: Parental Perspectives. J Family Psychother.

[63] Viggiano T, Pincus HA, Crystal S (2012). Care transition interventions in mental health. Curr Opin Psychiatry.

[64] Kalseth J, Lassemo E, Wahlbeck K, Haaramo P, Magnussen J. (2016). Psychiatric readmissions and their association with environmental and health system characteristics: a systematic review of the literature. BMC Psychiatry, 16(1). 10.1186/s12888-016-1099-8. 10.1186/s12888-016-1099-8 PMC5100223 27821155

[65] Joynt KE, Jha AK (2012). Thirty-Day Readmissions — Truth and Consequences. N Engl J Med.

[66] Brenes GA, Danhauer SC, Lyles MF, Hogan PE, Miller ME (2015). Barriers to Mental Health Treatment in Rural Older Adults. Am J Geriatric Psychiatry.

[67] Bocker E, Glasser M, Nielsen K, Weidenbacher-Hoper V. Rural older adults’ mental health: status and challenges in care delivery. Rural Remote Health. 2012;12(4):1–3. 23145784

[68] Weinberger (2009). Perceived barriers to mental health care and goal setting among depressed, community-dwelling older adults. Patient Prefer Adherence.

[69] Cook BL, Doksum T, Chen C, Carle A, Alegría M (2013). The role of provider supply and organization in reducing racial/ethnic disparities in mental health care in the U.S. Soc Sci Med.

[70] Stockdale SE, Tang L, Zhang L, Belin TR, Wells KB (2007). The Effects of Health Sector Market Factors and Vulnerable Group Membership on Access to Alcohol, Drug, and Mental Health Care. Health Serv Res.

[71] Wei W, Sambamoorthi U, Olfson M, Walkup JT, Crystal S (2005). Use of Psychotherapy for Depression in Older Adults. Am J Psychiatry.

[72] Benjenk I, Chen J (2019). Variation of Follow-Up Rate After Psychiatric Hospitalization of Medicare Beneficiaries by Hospital Characteristics and Social Determinants of Health. Am J Geriatric Psychiatry.

[73] Tyler N, Wright N, Waring J. Interventions to Improve Discharge from Acute Adult Mental Health Inpatient Care to the Community: Systematic Review and Narrative Synthesis. BMC Health Serv Res. 2019;19(1). 10.21203/rs.2.10436/v2. 10.1186/s12913-019-4658-0 PMC6876082 31760955

[74] Currie LB, Patterson ML, Moniruzzaman A, McCandless LC, Somers JM (2018). Continuity of Care among People Experiencing Homelessness and Mental Illness: Does Community Follow-up Reduce Rehospitalization?. Health Serv Res.

[75] General Hospital Psychiatry, 40, 55–59. 10.1016/j.genhosppsych.2016.01.004.

[76] The Journal of Clinical Psychiatry, 81(5). 10.4088/jcp.20m13344. 10.4088/JCP.20m13344 PMC10476606 32936543

[77] Interian A, Ang A, Gara MA, Rodriguez MA, Vega WA (2011). The long-term trajectory of depression among Latinos in primary care and its relationship to depression care disparities. Gen Hospital Psychiatr..

[78] Storm M, Siemsen IMD, Laugaland KA, Dyrstad DN, Aase K (2014). Quality in transitional care of the elderly: Key challenges and relevant improvement measures. Int J Integr Care.

[79] Storm M, Aase K, Waring J, Schibevaag L (2017). The role of professionals’ competencies in care transitions. Researching quality in care transitions: international perspectives.

[80] Carson NJ, Vesper A, Chen C, Lê Cook B (2014). Quality of Follow-Up After Hospitalization for Mental Illness Among Patients From Racial-Ethnic Minority Groups. Psychiatric Serv.

[81] Cook BL, McGuire TG, Alegría M, Normand S-L (2011). Crowd-out and Exposure Effects of Physical Comorbidities on Mental Health Care Use: Implications for Racial-Ethnic Disparities in Access. Health Serv Res.

[82] Conlon M, Tew J, Solai LK, Gopalan P, Azzam P, Karp JF (2020). Care Transitions in the Psychiatric Hospital: Focus on Older Adults. Am J Geriatric Psychiatry.

[83] Li H, Proctor E, Morrow-Howell N (2005). Outpatient mental health service use by older adults after acute psychiatric hospitalization. J Behav Health Serv Res.

[84] Alegría M, Alvarez K, Ishikawa RZ, DiMarzio K, McPeck S (2016). Removing Obstacles To Eliminating Racial And Ethnic Disparities In Behavioral Health Care. Health Aff.

[85] Ortiz G (2019). Predictors of 30-day Postdischarge Readmission to a Multistate National Sample of State Psychiatric Hospitals. J Healthc Qual.

[86] Puntis SR, Rugkåsa J, Burns T (2016). The association between continuity of care and readmission to hospital in patients with severe psychosis. Soc Psychiatry Psychiatr Epidemiol.

[87] Stahler GJ, Mennis J, Cotlar R, Baron DA (2009). The influence of neighborhood environment on treatment continuity and rehospitalization in dually diagnosed patients discharged from acute inpatient care. Am J Psychiatry.

[88] National Committee for Quality Assurance. State of Health Care Quality. 2012 Retrieved from https://www.ncqa.org/report-cards/health-plans/state-of-health-care-quality-report/.

[89] Donisi V, Tedeschi F, Salazzari D, Amaddeo F (2016). Pre- and post-discharge factors influencing early readmission to acute psychiatric wards: implications for quality-of-care indicators in psychiatry. Gen Hosp Psychiatry.

[90] Stahler GJ, Mazzella S, Mennis J, Chakravorty S, Rengert G, Spiga R (2007). The effect of individual, program, and neighborhood variables on continuity of treatment among dually diagnosed individuals. Drug Alcohol Depend.

[91] West AN, Weeks WB, Charlton ME (2015). Differences Among States in Rural Veterans’ Use of VHA and Non-VHA Hospitals. J Rural Health.

[92] Decker KP, Peglow SL, Samples CR, Cunningham TD (2017). Long-Term Outcomes After Residential Substance Use Treatment: Relapse, Morbidity, and Mortality. Mil Med.

[93] Zuehlke JB, Kotecki RM, Kern S, Sholty G, Hauser P (2016). Transformation to a recovery-oriented model of care on a veterans administration inpatient unit. Psychiatr Rehabil J.

[94] Bowersox NW, Saunders SM, Berger B (2012). Post-inpatient Attrition from Care “As Usual” in Veterans with Multiple Psychiatric Admissions. Commun Ment Health J.

[95] Meshberg-Cohen S, DeViva JC, Rosen MI (2017). Counseling Veterans Applying for Service Connection Status for Mental Health Conditions. Psychiatric Serv.

[96] Journal of the American Psychiatric Nurses Association, 25(3), 208–217. 10.1177/1078390318786024. 10.1177/1078390318786024 29973093

[97] College of Psychiatric and Neurologic Pharmacists (2015). 2015 Poster Abstracts. J Pharm Pract.

[98] Koval, R. D., Mcdonagh, J., Grubaugh, A., Young, W., Corcoran, B., Lee, A., … Edlund, B. Implementation of Recovery Programming on an Inpatient Acute Psychiatric Unit and Its Impact on Readmission. J Addictions Nurs. 2016; 27(2), 101–108. 10.1097/jan.0000000000000121. 10.1097/JAN.0000000000000121 27272994

[99] College of Psychiatric and Neurologic Pharmacists (2016). 2016 Poster Abstracts. J Pharm Pract.

[100] Wray AM, Hoyt T, Welch S, Civetti S, Anthony N, Ballester E, Tandon R (2019). Veterans Engaged in Treatment, Skills, and Transitions for Enhancing Psychiatric Safety (VETSTEPS). Psychiatr Rehabil J.

[101] College of Psychiatric and Neurologic Pharmacists (2011). 2011 Poster Abstracts. J Pharm Pract.

[102] Complementary Therapies in Medicine, 40, 42–47. 10.1016/j.ctim.2018.07.013.

[103] College of Psychiatric and Neurologic Pharmacists (2013). 2013 Poster Abstracts. J Pharm Pract.

[104] Pfeiffer PN, Ganoczy D, Zivin K, McCarthy JF, Valenstein M, Blow FC (2012). Outpatient Follow-Up After Psychiatric Hospitalization for Depression and Later Readmission and Treatment Adequacy. Psychiatric Serv.

[105] Sloan PA, Asghar-Ali A, Teague A, Body E, Kunik ME (2010). Psychiatric Hospitalists and Continuity of Care: A Comparison of Two Models. J Psychiatr Pract.

[106] Abrams TE, Vaughan-Sarrazin M, Vander Weg MW (2011). Acute Exacerbations of Chronic Obstructive Pulmonary Disease and the Effect of Existing Psychiatric Comorbidity on Subsequent Mortality. Psychosomatics.

[107] Health & Justice, 7(1). 10.1186/s40352-019-0099-4.

[108] Clinical Trials: Journal of the Society for Clinical Trials, 8(2), 196–204. 10.1177/1740774510392931.

[109] Kim HM, Pfeiffer P, Ganoczy D, Valenstein M (2011). Intensity of Outpatient Monitoring After Discharge and Psychiatric Rehospitalization of Veterans With Depression. Psychiatric Serv.

[110] Psychiatric Services, 60(4), 451–458. 10.1176/ps.2009.60.4.451.

[111] Spiegel D (1987). Family involvement in managing schizophrenia. Western J Med.

[112] National Conference of State Legislatures. Veteran Homelessness: Overview of State and Federal Resources. 2020. Retrieved February 22, 2021, from www.ncsl.org website: https://www.ncsl.org/research/military-and-veterans-affairs/veteran-homelessness-an-overview-of-state-and-federal-resources.aspx.

[113] Burton CZ, Abraham KM, Grindle CM, Visnic S, Hack SM, McCarthy JF, Bowersox NW (2018). Outreach to veterans with serious mental illness who are lost to care: Predictors of outreach contact. Psychol Serv.

[114] West JC, Rae DS, Mojtabai R, Duffy FF, Kuramoto J, Moscicki E, Narrow WE (2015). Planning Patient-Centered Health Homes for Medicaid Psychiatric Patients at Greatest Risk for Intensive Service Use. Commun Ment Health J.

[115] Bowersox NW. Treatment Attrition and Relapse Readmission in Psychiatric Inpatients: Predictors of Treatment Engagement and Psychiatric Relapse. 2009. Retrieved from https://epublications.marquette.edu/dissertations_mu/18/.

[116] Springer JR, Sloan PA, Benge JF, Spence M, Carlo I, Teng EJ (2009). From Dangerous to Discharged: An Application of Social-Learning-Based Procedures in an Acute Hospital Setting. Clin Case Stud.

[117] Moullin JC, Dickson KS, Stadnick NA, Rabin B, Aarons GA. Systematic review of the Exploration, Preparation, Implementation, Sustainment (EPIS) framework. Implement Sci. 2019; 14(1). 10.1186/s13012-018-0842-6. 10.1186/s13012-018-0842-6 PMC6321673 30611302

[118] Lengnick-Hall R, Willging C, Hurlburt M, Fenwick K, Aarons GA. Contracting as a bridging factor linking outer and inner contexts during EBP implementation and sustainment: a prospective study across multiple U.S. public sector service systems. Implement Sci. 2020;15(1). 10.1186/s13012-020-00999-9. 10.1186/s13012-020-00999-9 PMC7288508 32527274

[119] Health Resources & Services Administration. HRSA Accreditation and Patient-Centered Medical Home Recognition Initiative. 2018. Retrieved from Bureau of Primary Health Care website: https://bphc.hrsa.gov/qualityimprovement/clinicalquality/accreditation-pcmh/index.html.

[120] National Institute of Mental Health. PAR-18-429: Pilot Studies to Test the Initiation of a Mental Health, Family Navigator Model to Promote Early Access, Engagement and Coordination of needed Mental Health Services for Children and Adolescents (R34-Clinical Trial Required). 2017 Retrieved from grants.nih.gov website: https://grants.nih.gov/grants/guide/pa-files/PAR-18-429.html.

[121] Halsall T, Manion I, Iyer SN, Mathias S, Purcell R, Henderson J (2019). Trends in mental health system transformation: Integrating youth services within the Canadian context. Healthc Manage forum.

[122] Sharma A, Sharma SD, Sharma M (2017). Mental health promotion: a narrative review of emerging trends. Curr Opin Psychiatry.

[123] Administration and Policy in Mental Health and Mental Health Services Research, 46(2), 154–166. 10.1007/s10488-018-0901-y. 10.1007/s10488-018-0901-y 30353419

[124] Barling J, Loughlin C, Kelloway EK (2002). Development and test of a model linking safety-specific transformational leadership and occupational safety. J Appl Psychol.

[125] Zohar D (2002). Modifying supervisory practices to improve subunit safety: A leadership-based intervention model. J Appl Psychol.

[126] Hong Y, Liao H, Hu J, Jiang K (2013). Missing link in the service profit chain: A meta-analytic review of the antecedents, consequences, and moderators of service climate. J Appl Psychol.

[127] Miech EJ, Rattray NA, Flanagan ME, Damschroder L, Schmid AA, Damush TM (2018). Inside help: An integrative review of champions in healthcare-related implementation. SAGE Open Medicine.

[128] BMJ Evidence-Based Medicine, 24(3), 103–108. 10.1136/bmjebm-2018-111019.

[129] Bryan JL, Kauth MR, Asghar-Ali AA (2019). Transforming Veterans Health Administration Mental Health Clinician Education and Practices. J Continuing Educ Health Professions.

[130] Timko C, Schultz NR, Britt J, Cucciare MA (2016). Transitioning From Detoxification to Substance Use Disorder Treatment: Facilitators and Barriers. J Subst Abuse Treat.

[131] Wittink H, Oosterhaven J (2018). Patient education and health literacy. Musculoskelet Sci Pract.

[132] Keogh B, Callaghan P, Higgins A (2015). Managing preconceived expectations: mental health service users experiences of going home from hospital: a grounded theory study. J Psychiatr Ment Health Nurs.

[133] Henderson, J. L., Cheung, A., Cleverley, K., Chaim, G., Moretti, M. E., de Oliveira,C., … Szatmari, P. Integrated collaborative care teams to enhance service delivery to youth with mental health and substance use challenges: protocol for a pragmatic randomised controlled trial. BMJ Open. 2017;7(2):e014080. 10.1136/bmjopen-2016-014080 PMC5293997 28167747

[134] Baldwin SM, Zook S, Sanford J (2018). Implementing Posthospital Interprofessional Care Team Visits to Improve Care Transitions and Decrease Hospital Readmission Rates. Prof Case Manage.

[135] General Hospital Psychiatry, 39, 59–65. 10.1016/j.genhosppsych.2015.11.002.

